# Prevalence of chronic diseases by immigrant status and disparities in chronic disease management in immigrants: a population-based cohort study, Valore Project

**DOI:** 10.1186/1471-2458-13-504

**Published:** 2013-05-24

**Authors:** Alessandra Buja, Rosa Gini, Modesta Visca, Gianfranco Damiani, Bruno Federico, Paolo Francesconi, Daniele Donato, Alessandro Marini, Andrea Donatini, Salvatore Brugaletta, Vincenzo Baldo, Mariadonata Bellentani

**Affiliations:** 1 Department of Molecular Medicine, Laboratory for Public Health and Population Studies, University of Padova, Via Loredan 18, Padova 35128, Italy; 2 Agenzia regionale di sanità della Toscana, Florence, Italy; 3 Agenas, Agenzia Nazionale per i Servizi Sanitari, Rome, Italy; 4 Facoltà di Medicina, Università Cattolica Sacro Cuore di Roma, Rome, Italy; 5 Facoltà di Scienze Motorie, Università degli studi di Cassino, Cassino, Italy; 6 ULSS 16 Padova, Regione Veneto, Padova, Italy; 7 Zona Territoriale Senigallia, Regione Marche, Ancona, Italy; 8 Regione Emilia Romagna, Bologna, Italy; 9 ASP 7 Ragusa, Regione Sicilia, Ragusa, Italy

## Abstract

**Background:**

For chronic conditions, disparities can take effect cumulatively at various times as the disease progresses, even when care is provided. The aim of this study was to quantify the prevalence of diabetes, congestive heart failure (CHF) and coronary heart disease (CHD) in adults by citizenship, and to compare the performance of primary care services in managing these chronic conditions, again by citizenship.

**Methods:**

This is a population-based retrospective cohort study on 1,948,622 people aged 16 years or more residing in Italy. A multilevel regression model was applied to analyze adherence to care processes using explanatory variables at both patient and district level.

**Results:**

The age-adjusted prevalence of diabetes was found higher among immigrants from high migratory pressure countries (HMPC) than among Italians, while the age-adjusted prevalence of CHD and CHF was higher for Italians than for HMPC immigrants or those from highly-developed countries (HDC). Our results indicate lower levels in all quality management indicators for citizens from HMPC than for Italians, for all the chronic conditions considered. Patients from HDC did not differ from Italian in their adherence to disease management schemes.

**Conclusion:**

This study revealed a different prevalence of chronic diseases by citizenship, implying a different burden of primary care by citizenship. Our findings show that more effort is needed to guarantee migrant-sensitive primary health care.

## Background

Access to health care (in the sense of the timely use of personal health care services to achieve the best health outcomes) is an essential prerequisite for ensuring good-quality health care and improving quality of life, and life expectancy [1]. From the perspectives of European integration and human rights, the health of migrants and their access to health care are important health policy issues [2,3]. Immigration is a phenomenon that has increased considerably in Italy in the last decade: foreign residents numbered 356,000 in 1991 (0.63% of the population), while they totaled 1.3 million (2.3%) in 2001, meaning a fourfold increase in ten years. By 2008, there were 3,432,651 regular immigrants registered in Italy, accounting for 6.5% of the population [4]. Clear legislation was enacted as of 1998 to regulate the immigrant population’s access to services provided under the Italian national health system (NHS). The declared objective is to allow legal immigrants access to health services on a par with Italian citizens [5]. The Italian NHS is funded mainly through taxes and provides universal access to health services. General practitioners (GPs) act as gatekeepers to the health system. Regular immigrants register with the NHS in the same way as Italian citizens they have access to: primary care, ambulatory care, hospital care, rehabilitation and emergency care [6]. In recent years, a growing body of literature has focused on whether there are disparities in how immigrants and the autochthonous population access the health services, even though the universality of the health benefits for virtually everyone in the country is assured. After controlling for individual characteristics, Giannoni [7] found that foreigners were less likely to access healthcare services than Italian citizens. Another report [8] indicated that foreigners and foreign-born people suffer from unequal access to health care services; they are more likely to contact emergency services and less likely to visit specialist doctors or use preventive care, while no differences emerged in their use of primary care. More detailed information is needed to assess migrants’ use of primary health care services with a view to monitoring and improving their health, and assuring appropriate and accessible primary health care services to this portion of the population [9]. In fact, primary care is the first interface between individuals, families and communities, and the NHS, bringing health care as close as possible to where people live and work, and it is this level of care that ensures a reducing in health disparities across population subgroups [10]. Furthermore the need for better data on migrant health issues has also been recognized in the World Health Organization (WHO) resolution on the health of migrants [11].

Worldwide, there is currently a growing interest in redesigning health care organizations, and primary care in particular, to improve the quality of care for chronic diseases (which have overtaken infectious diseases as the leading cause of death and disability) and to guarantee their equitable management. In chronic conditions, disparities can take effect cumulatively at various times as the disease progresses: in its genesis, or when it is still only latent (in terms of exposure to risk factors), and after it has been recognized (in the expression of the demand for), as well as when care is provided (in the diagnostic treatment and monitoring process). Such disparities lead to health inequalities that affect the prevalence of a chronic disease and its negative outcomes and related mortality rates.

The aim of the present study was: (i) to investigate the prevalence of certain major chronic diseases, i.e. diabetes, coronary heart disease (CHD), and congestive heart failure (CHF), by citizenship; and (ii) to compare Italian and non-Italian citizens’ adherence to disease management guidelines, based on the rationale that measuring adherence could shed light on whether physicians achieve the same degree of patient involvement in the management of disease for immigrants as for the Italian population.

## Methods

Italy is divided administratively into 20 regions, and each regional government is responsible for fulfilling the objectives of the National Health Plan in its area. These regional authorities plan and organize health care facilities and activities through their regional health departments. They also coordinate and control local health units (LHU), each of which is a separate NHS unit that plans and delivers health care services to its local community. Each LHU is organized into geographical subareas called Health Districts (HD), which manage all the local primary care structures and community services. In Italy, all residents registered with the NHS (be they Italians or regular immigrants) are listed with a general practitioner (GP) of their choice. GPs have a gate-keeping role. Exemptions from prescription charges for drugs and appropriate diagnostic tests (specific to each chronic condition) are currently applicable to patients with certain chronic conditions, as established in a Decree of the Ministry of Public Health approved in 1999. Such patients need to exhibit their GP’s prescription to obtain exempted drugs and diagnostic tests.

### Data and variables

Six Italian regions, i.e. two in northern Italy (Lombardy and Veneto), three in central Italy (Emilia Romagna, Tuscany and Marche), and one in southern Italy (Sicily) took part in the VALORE project, a scheme organized by the National Agency for Regional Health Systems to assess the association between quality of care for chronic diseases and the organization of primary care [12]. The present study was an offshoot of this project, involving one or two LHUs from each region (8 in all) and 2–4 HDs for each LHU (21 in all), which shared their data; each regional system chose which LHUs and HDs to enroll in the study. The dataset used in our analysis was produced by automatically processing administrative records. The number of individuals aged 16 years or more as at 1 January 2008 amounted to 1,948,622; the cases of diabetes, CHD or CHF were identified by means of algorithms developed by the Tuscany Regional Public Health Agency, based on the diagnoses indicated in the hospital discharge records or on disease-specific drug-dispensing records, or disease-specific health care copayment exemptions.

This procedure enabled us to identify 105,987 patients with diabetes, 86,725 with CHD, and 28,062 with CHF, who formed the samples of patients considered in this study.

Process indicators, which were used to assess what the health care provider did for a given patient and how well it was done [13], were chosen among those identified and defined by scientific associations as quality measures for improving patient outcomes.

In particular, for the diabetics we measured three indicators that the OECD [14] considers indicative of the quality of care for diabetes at health system level, i.e.: annual HbA1c testing; annual screening for nephropathy; annual LDL cholesterol testing. These indicators were calculated in terms of: the percentage of patients who had one or more HbA1c test a year; the percentage of patients who had at least one microalbuminuria test during the year considered; the percentage of patients who had at least one annual LDL cholesterol test.

For cases of CHD, we measured three indicators that the Chronic Stable Coronary Artery Disease Work Group [15] considers as quality measures of care for CHF for the purpose of improving outcomes for outpatients with chronic stable coronary artery disease, i.e.: therapy with angiotensin-converting enzyme (ACE) inhibitors; therapy with anti-thrombotic agents; and annual total cholesterol monitoring. These indicators were calculated in terms of: the percentage of patients who had at least two prescriptions of ACE inhibitors in the same year, separated by an interval of at least of 180 days; the percentage of patients with at least two prescriptions of anti-thrombotic agents, separated by an interval at least 180 days; the percentage of patients with at least one annual total cholesterol test.

For patients with CHF, we chose four indicators that the Heart Failure Work Group [16] considers indicative of the quality of care for CHF for the purposes of improving outcomes for outpatients with heart failure, i.e.: therapy with ACE inhibitors; therapy with beta-blockers; 6-monthly monitoring of creatinine, and Na and K; and annual echocardiography. These indicators were calculated in terms of: the percentage of patients with at least two prescriptions of ACE inhibitors in a year, separated by an interval of at least 180 days; the percentage of patients with at least two prescriptions of beta-blockers, separated by an interval of at least 180 days; the percentage of patients with at least one creatinine, Na and K test in the previous six months; and the percentage of patients with at least one annual echocardiogram.

We enrolled people registered with the NHS (all Italian citizens and regular immigrants), classifying their nationality as follows: Italians; immigrants from highly-developed countries (HDC); and immigrants from high migratory pressure countries (HMPC) [17]. The HMPC included new Member States of the European Union, countries in Central-Eastern Africa, Asia (except for Israel and Japan), and Central and South America; by extension, stateless individuals were also included in this group. The HDC included the other European countries, North America, Oceania, Israel and Japan.

The Charlson index was calculated to assess patients’ comorbidities. It has been demonstrated that this index is a valid and reliable method for measuring comorbidity suitable for use in clinical research and, although it was developed and validated in hospitalized patients, it has since been adapted and validated for primary care and community populations [18].

Subjects whose citizenship was not known were excluded from the analysis (this applied to 5.6% of the diabetics and CHD patients, and 4.78% of the CHF patients).

### Statistical methods

The data were summarized as numbers (percentages) of subjects for categorical variables. A multilevel logistic regression model was applied to analyze the association between nationality and adherence to standards of care. The data had a hierarchical structure, with the patient as the first level and the HD as the second level. The dependent variables were analyzed in dichotomous form (yes/no) for each patient’s adherence to evidence-based quality of care requirements for managing the diseases considered. Besides nationality, the independent variables were: gender, age band (16–44, 45–64, 65–74, 75–84, >85 years), time since diagnosis (dichotomized as ≤3 y and >3 y), and the Charlson index on the first level of the hierarchical structure, and HD on the second level.

The study complies with the Helsinki Declaration and with Italian privacy law (Decree No. 196/2003). In addition, resolution n. 85/2012 of the Italian Guarantor for the protection of personal data recently confirmed permission to process personal data for medical, biomedical and epidemiological research; data concerning health status can be used in aggregated form for the purpose of scientific studies [19]. No identifiable personal details were used for this study. The dataset used in the study is not available to the public. Approval for the use of encrypted and aggregated data was also obtained from the Italian College of General Practitioners.

## Results

The estimated overall crude prevalence of diabetes was 5.43% (95% CI 5.33-5.54); the age-standardized prevalence amounted to 5.45% (95% CI 5.42 -5.48) among Italian citizens, 3.83% (95% CI 3.80-3.86) among immigrants from HDC, and 6.96% (95% CI 6.93-7.00) among HMPC immigrants.

The estimated overall crude prevalence of CHD was 4.20% (95% CI 4.17-4.23); the age-standardized prevalence amounted to 4.52% (95% CI 4.49-4.55), 3.05% (95% CI 3.03-3.08) and 3.78% (95% CI 3.75-3.81), respectively, for the Italian, HDC and HMPC citizens.

The estimated overall crude prevalence of CHF was 1.44% (95% CI 1.42 -1.46), with an age-standardized prevalence of 1.48% (95% CI 1.46-1.49), 1.12% (95% CI 1.10-1.13) and 1.19% (95% CI 1.17-1.20), respectively, in the Italian, HDC and HMPC groups.

Considering the diabetics as a whole, HbA1c tests had been conducted in 60.50% of cases, LDL cholesterol tests in 57.50%, and creatinine in 63.27%, while only 44.19% of the diabetic patients had undergone a comprehensive assessment including all three blood tests. Overall, 57.35% of the CHD patients were being treated with ACE inhibitors and 61.91% with antithrombotic therapy; LDL cholesterol tests were performed in 54.01%. As for the CHF patients, 55.46% were treated with ACE inhibitors and 36.73% with beta-blockers; 56.45% were tested for Na and K, and only 15.45% had undergone echocardiography.

The results of our multilevel logistic regressions (Tables 1, 2, 3) showed marked differences in all quality management indicators for the three chronic diseases considered. By comparison with their Italian counterparts, people from HMPC with diabetes had more than 30% lower odds of annual glycated hemoglobin testing, 29% lower odds of annual renal function tests, and 45% lower odds of having their cholesterol profile checked, whereas diabetics from HDC did not differ from Italian diabetics in terms of their adherence to disease management schemes. Patients from HMPC with CHD had a more than 40% lower odds of being treated with ACE inhibitors and anti-thrombotic agents, and were only half as likely as Italians to have their cholesterol profile checked. Here again, CHD patients from HDC did not differ from native Italians in their adherence to disease management schemes (apart from the use of ACE inhibitor therapy). CHF patients from HMPC had almost 70% lower odds of being treated with ACE inhibitors, 50% lower odds when it came to beta-blockers, a ≈ 40% lower odds of having their creatinine, Na and K checked, and ≈ 30% lower odds of undergoing echocardiography, while CHF patients from HDC did not differ from Italian citizens in their adherence to disease management programs.

**Table 1:** Multilevel logistic regressions in diabetics (dependent variable: adherence to disease management quality indicators)

		**OR**	**95%LL**	**95%UL**	***p***
**Annual HbA1c testing**					
Nationality (Italian = reference)	HDC	0.95	0.75	1.21	0.69
	HMPC	0.64	0.59	0.69	0.00
**Annual screening for nephropathy**					
Nationality (Italian = reference)	HDC	1.02	0.80	1.30	0.90
	HMPC	0.71	0.66	0.77	0.00
**Annual LDL cholesterol testing**					
Nationality (Italian = reference)	HDC	0.95	0.75	1.20	0.67
	HMPC	0.55	0.50	0.60	0.00

All regressions adjusted for gender, age band (16–44, 45–64, 65–74, 75–84, > 85 years), time since diagnosis (dichotomized as ≤3y and >3y), and the Charlson index on a first level, and health districts on a second level.

**Table 2:** **Multilevel logistic regressions in CHD patients** (**dependent variable**: **adherence to disease management quality indicators**)

		**OR**	**95%LL**	**95%UL**	***p***
**Therapy with ACE inhibitors**					
Nationality (Italian = reference)	HDC	0.73	0.55	0.97	0.03
	HMPC	0.58	0.50	0.67	0.00
**Therapy with anti**-**thrombotic agents**					
Nationality (Italian = reference)	HDC	1.02	0.76	1.39	0.86
	HMPC	0.59	0.50	0.68	0.00
**Annual total cholesterol monitoring**					
Nationality (Italian = reference)	HDC	0.84	0.63	1.12	0.23
	HMPC	0.47	0.40	0.54	0.00

All regressions adjusted for gender, age band (16–44, 45–64, 65–74, 75–84, > 85 years), time since diagnosis (dichotomized as ≤3y and >3y), and the Charlson index on a first level, and health districts on a second level.

**Table 3:** **Multilevel logistic regressions in CHF patients** (**dependent variable**:**adherence to disease management quality indicators**)

		**OR**	**95%LL**	**95%UL**	***p***
**Therapy with ACE inhibitors**					
Nationality (Italian = reference)	HDC	1.37	0.80	2.32	0.25
	HMPC	0.34	0.26	0.45	0.00
**Therapy with beta**-**blockers**					
Nationality (Italian = reference)	HDC	0.87	0.52	1.45	0.59
	HMPC	0.50	0.37	0.66	0.00
**Creatinine**, **Na and K monitoring**					
Nationality (Italian = reference)	HDC	1.04	0.64	1.70	0.87
	HMPC	0.57	0.44	0.76	0.00
**Annual echocardiogram**					
Nationality (Italian = reference)	HDC	1.01	0.55	1.86	0.96
	HMPC	0.68	0.48	0.97	0.03

All regressions adjusted for gender, age band (16–44, 45–64, 65–74, 75–84, > 85 years), time since diagnosis (dichotomized as ≤3y and >3y), and the Charlson index on a first level, and health districts on a second level.

## Discussion

To the best of our knowledge, this is the first study to be conducted in Europe on equity in access to health care services for the management of chronic diseases, by migrant status. An earlier article [20] claimed that the EU countries have a relatively weak system for assessing the needs and planning the health care of their migrant and ethnic minority populations. The lack of relevant data on cardiovascular diseases (CVD) and diabetes across the EU needs to be urgently addressed. Our findings show that the age-standardized prevalence of CHD and CHF is significantly lower in immigrants than in Italian citizens, probably due to a ‘healthy immigrant effect’. But the age-standardized prevalence of diabetes is significantly higher among HMPC immigrants than for Italian citizens, probably due to ethnic differences in the prevalence of this disease, as previously documented in the literature [21,22].

Our study found inequalities in diabetes management by citizenship, and particularly that diabetic patients from HMPC were far less likely than Italian patients to score well in all three indicators of the quality of their disease’s management. An Irish study [23] on disparities in the management of diabetics by immigrant status also showed that immigrants had a worse glycemic control and were less likely to monitor their blood sugar levels daily. A number of American studies [24,25] identified mainly racial disparities and ethnic differences in the quality of diabetes care, but the immigration phenomena and health care system in the US differ considerably from those of the EU: the US population includes many longstanding immigrants and research has focused more on inequalities by ethnic minority group rather than by migrant status.

As for the disparities in the management of immigrants with cardiovascular diseases, one study found immigrants more likely than patients born in the US to be unaware of their CVD risk factors, and consequently less motivated to seek treatment or modify their behavior to prevent negative CVD outcomes [26]. This is consistent with our finding that immigrants from HMPC with CHD or CHF fared less well in all management indicators and were up to 70% less likely to be taking ACE inhibitors than their Italian counterparts.

Migrants may experience language difficulties, issues concerning their cultural beliefs, and problems with obtaining transportation, time off work, and child care. They may also be less attentive to their health, as well as experiencing cultural barriers to health care (there are no financial barriers in Italy because chronically ill patients are exempted from charges for routine tests). All of these aspects might interfere with how well their chronic diseases are managed. Newly-arrived migrants may also be unfamiliar with how the health care system works in their new place of residence. Access to health services and their appropriate use are often influenced by people’s unfamiliarity with enrolment processes and entry points, and structural barriers to receiving care, and discouraging or discriminatory treatment by health care staff. Even when migrants do access health services, they may find it difficult to explain their symptoms or understand instructions they receive for their treatment [27].

Judging from our findings, action is needed to reduce disparities, targeting strategies for the immigrant population. Until now, a “waiting paradigm” has been adopted in the Italian approach to primary care, which means waiting for an event to take action on, a problem to solve. Waiting is the classical health care paradigm of the biomedical model, and it has also become the dominant paradigm in territorial and primary care [28]. One way to reduce the inequalities identified in the management of chronic conditions would be to adopt a more proactive approach to primary care, seeking to identify patients’ needs without waiting for them to come forward. A model tending to provide a proactive care is particularly important when it comes to managing chronic diseases, and part of the empowerment philosophy includes interactive teaching strategies designed to involve patients in solving their problems, addressing their cultural and social needs, making them take some responsibility for their daily care, and encouraging them to monitor their risk factors and complications regularly [29]. Such a model had never been applied in Italy up until the time to which the data analyzed in the present study refer; more recently, it has been applied by a few LHUs. Further research will be useful to assess whether it can reduce chronic disease management inequalities by citizenship.

Several other aspects could be addressed to improve migrants’ access to quality health care [30], e.g. extending the hours when the services are provided, the case management of patients with chronic diseases [31]. One review [32] designed to identify the action taken by health systems to improve care for diabetes among the socially disadvantaged found that cultural tailoring of intervention, community educators, one-on-one interventions with customized assessments and reassessments, and high-intensity interventions (>10 contact times) delivered over a lengthy period of time improved diabetes care among socially disadvantaged populations.

Our study has some limitations. First of all, not all the relevant socio-economic factors (e.g. level of formal education) were available in the database. In addition, only care process measures were assessed, and it is debatable whether better care processes are genuinely linked to an improvement in intermediate or final outcomes for patients [33,34]. On the other hand, a recent study on diabetics [35] found that those receiving the worst quality of care, as measured by process quality-of-care indicators based on screening guidelines, were at higher risk of all-cause mortality and cardiovascular morbidity than patients receiving the highest process quality of care. The strength of our study lies in that it was conducted on an unrestricted and unselected population of primary care patients, thus enabling an estimation of the prevalence of the diseases of interest and of the related primary care performance measures. On the other hand, these data could be biased due to an opportunistic sample of LHUs being enrolled by the regional systems. This important methodological issue was addressed by a recent paper on the consistency of the Valore database [12] used in the present study vis-à-vis other sources of data, such as primary care medical records and national surveys: the prevalence estimates of ischemic heart disease and treated diabetes were consistent across the regions between the Valore database and clinical primary care databases; for heart failure, the Valore estimates were systematically higher than GPs’ estimates in all five regions considered, the highest difference being 1.4% vs 1.1%. Finally, some subjects involved in the present study were lost to follow-up, as is usually the case in cohort studies. Drop-out from the cohort was recorded for around 4% of diabetics (and slightly more for non-Italian patients), and for around 6% and 10% of patients with CHD and CHF, respectively (with lower percentages for non-Italian patients). This happened when patients either died or moved away during the one-year follow-up. Loss to follow-up therefore should not have affected the direction of the disparity identified, but it has to be said that non-Italian patients might decide to leave the area without informing the LHU (we are unable to quantify the effect of this, but it is unlikely to be able to justify the differences we measured).

## Conclusion

In conclusion, our study shows that management indicators are useful not only for the overall evaluation of the care process for a given disease, but also to monitor disparities in the provision of health care services. Based on such indicators, the present study revealed numerous opportunities for improving the health care and management of immigrants from high migratory pressure countries. Our findings disclose citizenship-related inequalities in the provision of primary care for the chronic diseases investigated (diabetes, CHD and CHF). It was particularly evident that diabetic and cardiopathic patients from HMPC were far less likely than Italian patients to score well in all indicators of the quality of their disease’s management. This means that more effort is needed to guarantee migrant-sensitive primary health care systems.

## Competing interests

The authors have no relevant conflicts of interest to disclose.

## Authors’ contributions

Authorship: guarantors for the integrity of the study: AB statistical analysis and manuscript writing; RG participated in study design and coordination, performed data management; DG data interpretation and study conception; MV study conception and involvement of study centers; FB data interpretation and helped to draft the manuscript; DD participated in the study coordination with responsibility of the Veneto data and revision of the manuscript; MA participated in the study coordination with responsibility of the Marche data and revision of the manuscript; DA participated in the study coordination with responsibility of the Emilia Romagna data and helped to draft the manuscript and revision of the manuscript; BS participated in the study coordination with responsibility of the Sicily data and helped to draft the manuscript and revision of the manuscript; VB conceived of the study; BMD participated in the study design and coordination. All authors read and approved the final manuscript.

## Valore group

Antonio Brambilla, Massimiliano Correani, Nunziata Cosentino, Claudio Cricelli, Selene Fulvi, Pietro Gallina, Alessandro Marini, Giampiero Mazzaglia, Giuseppe Noto, Federica Palumbo, Alessandro Pasqua, Daniele Romeo, Renato Rubin, Stefano Sforza, Giulia Silvestrini, Eleonora Verdini, Giancarlo Viola

## Pre-publication history

The pre-publication history for this paper can be accessed here:

http://www.biomedcentral.com/1471-2458/13/504/prepub
